# Safety assessment of sulfasalazine: a pharmacovigilance study based on FAERS database

**DOI:** 10.3389/fphar.2024.1452300

**Published:** 2024-09-12

**Authors:** Wangyu Ye, Yuan Ding, Meng Li, Zhihua Tian, Shaoli Wang, Zhen Liu

**Affiliations:** Guang’anmen Hospital, China Academy of Chinese Medical Sciences, Beijing, China

**Keywords:** sulfasalazine, FDA adverse events reporting system, adverse drug reaction, adverse event, pharmacovigilance

## Abstract

**Background:**

Sulfasalazine is a widely used anti-inflammatory medication for treating autoimmune disorders such as ulcerative colitis (UC), Crohn’s disease, and rheumatoid arthritis. However, its safety profile has not been systematically evaluated in real-world settings. By analyzing the FDA Adverse Event Reporting System (FAERS) database, we identified risk signals associated with adverse reactions to sulfasalazine, offering valuable insights for clinical decision-making and risk management.

**Methods:**

Reports of adverse events (AEs) associated with sulfasalazine, covering the period from Q1 2004 to Q4 2023, were extracted from the FAERS database. Detailed case information was aggregated to assess demographic characteristics. The associations between sulfasalazine and adverse events were evaluated using the Proportional Reporting Ratio (PRR), Reporting Odds Ratio (ROR), Bayesian Confidence Propagation Neural Network (BCPNN), and Empirical Bayes Geometric Mean (EBGM).

**Results:**

We extracted 7,156 adverse event reports from the FAERS database where sulfasalazine was identified as the “Primary Suspect (PS)” drug. Using disproportionality analysis, we identified 101 preferred terms (PT) related to sulfasalazine across 24 organ systems. Notable adverse reactions consistent with the drug’s labeling were observed, including Stevens-Johnson syndrome, agranulocytosis, eosinophilic pneumonia, and crystalluria. Additionally, novel positive signals not previously documented in the drug label were identified, including acute febrile neutrophilic dermatosis, aseptic meningitis, glomerulonephritis, and hepatosplenic T-cell lymphoma.

**Conclusion:**

Most of the adverse reaction findings in this study are consistent with previous clinical research, and we have also identified new potential AEs associated with sulfasalazine. These findings provide valuable insights for the safety monitoring and clinical application of sulfasalazine.

## 1 Introduction

Sulfasalazine is a widely used anti-inflammatory and immunomodulatory medication, composed of sulfapyridine and 5-aminosalicylic acid (5-ASA), linked by an azo bond that imparts distinctive pharmacological properties (Azadkhan et al., 1982). In the intestine, sulfasalazine is poorly absorbed but undergoes enzymatic cleavage by azoreductases from intestinal bacteria, producing the active constituents sulfapyridine and 5-ASA (Das and Dubin, 1976). Sulfapyridine primarily exerts systemic anti-inflammatory effects by suppressing the synthesis of inflammatory mediators, reducing leukocyte infiltration at inflammatory sites, and modulating cytokine secretion (Hoult, 1986). In contrast, 5-ASA offers localized protection to the intestinal mucosa, potentially through mechanisms such as scavenging oxidative free radicals, inhibiting neutrophil infiltration, and preserving mucosal barrier integrity. The combined action of these components endows sulfasalazine with dual systemic and localized therapeutic efficacy (Nissim-Eliraz et al., 2021; Pruzanski et al., 1997; Joshi et al., 2005).

In clinical practice, sulfasalazine is widely used to manage rheumatic diseases such as rheumatoid arthritis, ankylosing spondylitis, and inflammatory bowel diseases (IBD) (Chen et al., 2014; Rains et al., 1995). Rigorous randomized controlled trials and case studies have validated sulfasalazine’s efficacy in significantly alleviating symptoms such as joint swelling, pain, and intestinal mucosal damage (Suarez-Almazor et al., 2000; Lim et al., 2016). Recent research has also explored its potential use in other autoimmune conditions, including psoriasis and alopecia areata. However, these applications require further validation through evidence-based studies (Aghaei, 2008; Menter et al., 2009). In summary, due to its unique chemical structure and diverse mechanisms of action, sulfasalazine has become a pivotal therapeutic option for managing chronic inflammation and autoimmune disorders.

In recent years, despite the emergence of biologics and targeted therapies that offer more effective treatments for autoimmune diseases (Davis and Ballas, 2017), sulfasalazine remains a mainstay in clinical practice due to its cost-effectiveness and proven efficacy (Lichtenstein et al., 2018). However, the unavoidable adverse reactions associated with sulfasalazine should not be overlooked. The primary cause is its metabolite, sulfapyridine, which frequently leads to toxic reactions characteristic of sulfonamide drugs, such as headaches, nausea, vomiting, and various allergic responses, often manifesting within the first few months of treatment (Rains et al., 1995; Taffet and Das, 1983; Navarro and Hanauer, 2003). Less common adverse effects include hematological abnormalities, hepatic impairment, pulmonary complications, and hypersensitivity reactions (Ransford and Langman, 2002). Long-term use of sulfasalazine may also reduce sperm count and motility, potentially leading to infertility (Bermas, 2020).

Even under stringent controls, clinical trials often fail to accurately predict the true risks encountered by patients in real-world clinical settings. Therefore, leveraging data mining from the FAERS is crucial, as it provides a wealth of real-world data on patient medication use and adverse reactions (Alomar et al., 2020). This study aims to analyze adverse reaction reports related to sulfasalazine in the FAERS database, using signal detection methodologies to identify potential adverse drug signals and provide essential insights for drug safety evaluations.

## 2 Methods

### 2.1 Data source

The FAERS database is used by the United States FDA to monitor adverse events related to drugs and therapeutic products. This database includes adverse event information reported by patients, healthcare professionals, and manufacturers. The primary purpose of FAERS is to identify potential safety signals and assist the FDA in evaluating and monitoring drug safety. A retrospective analysis was conducted using the publicly accessible FAERS database (https://fis.fda.gov/extensions/FPD-QDE-FAERS/FPD-QDE-FAERS.html) to examine adverse reaction reports associated with sulfasalazine. Adverse reaction reports related to sulfasalazine from Q1 2004 to Q4 2023 were extracted from the FAERS database and imported into R Studio 4.3.3 for comprehensive extraction, organization, and analysis.

### 2.2 Data processing and standardization

To ensure data accuracy and reliability, we extracted the most recent report for each case based on the case ID, retaining only the latest report and discarding earlier versions. We standardized drug names in the reports using the Medex_UIMA_1.3.8 system. We mapped both generic and brand names of drugs to a uniform standardized name to eliminate variability. We extracted reports related to sulfasalazine labeled as “Primary Suspect” to create our research dataset. During data processing, we classified adverse events (AEs) using preferred terms (PTs) according to the Medical Dictionary for Regulatory Activities (MedDRA, version 26.1) to ensure consistent terminology. We also categorized adverse events based on the System Organ Class (SOC). We collected information from sulfasalazine-related adverse event reports, including patient age, sex, reporter type, report time, and outcomes (e.g., death, hospitalization, life-threatening events), for descriptive statistical analysis.

### 2.3 Signal detection and analysis

**FIGURE 1:**
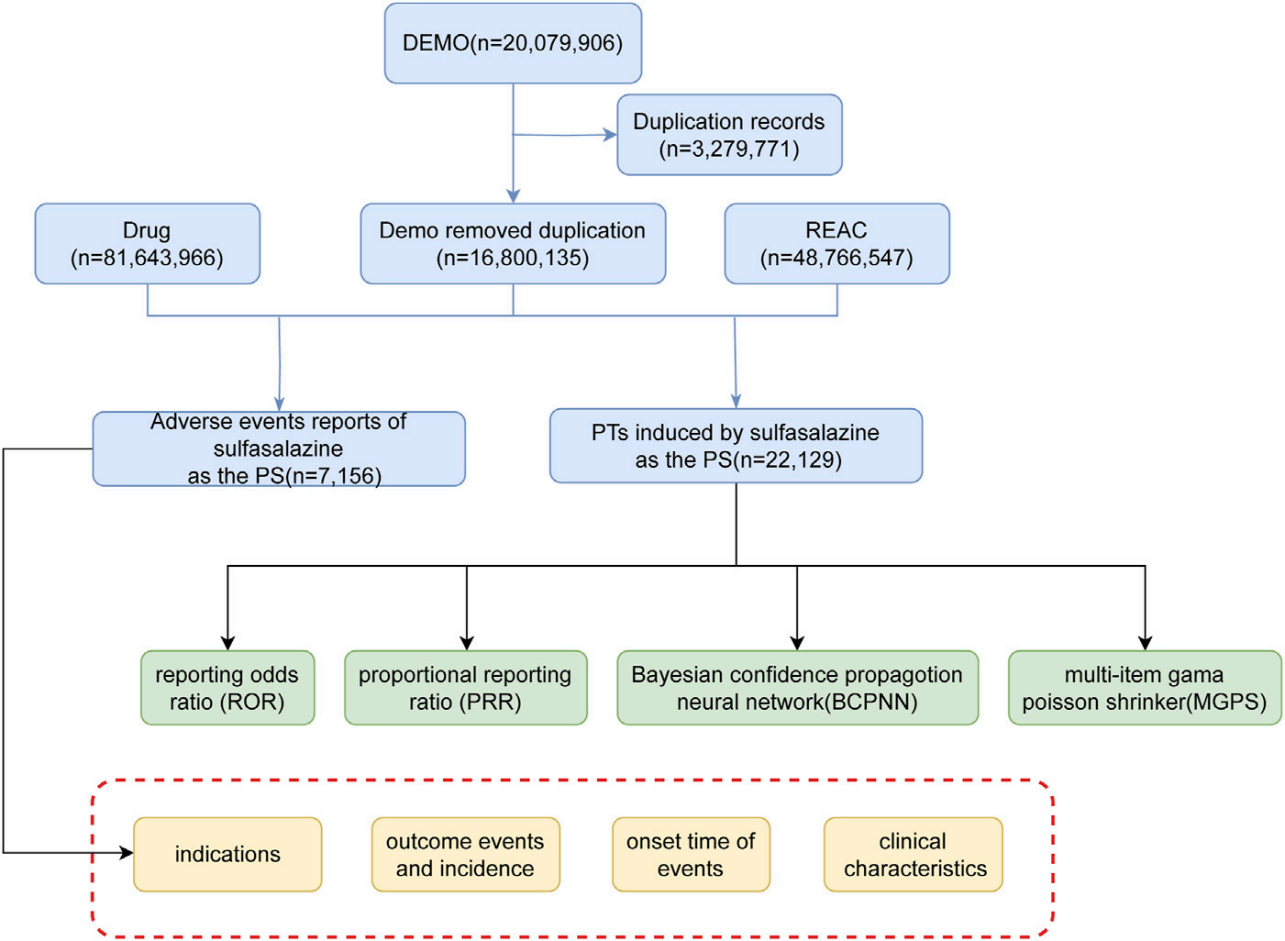
The flow diagram of selecting sulfasalazine-related AEs from FAERS database.

Disproportionality analysis is a data mining technique used to determine if there is an abnormal association between a specific drug and the adverse reactions reported. Disproportionality analysis is a commonly used method to evaluate the association between drugs and AEs. Its core principle involves comparing the observed frequencies of adverse events in exposed versus non-exposed groups using a contingency table, thereby quantifying the association between drugs and AEs (Supplementary Table S1). Our study utilized primary methods including the Proportional Reporting Ratio (PRR), Reporting Odds Ratio (ROR), Bayesian Confidence Propagation Neural Network (BCPNN), and Empirical Bayes Geometric Mean (EBGM) to assess the association between the exposed drug and AEs. A positive signal for adverse reactions is indicated if the lower limit of the 95% confidence interval (CI) for the ROR is greater than one and there are at least three reports of the AE. A significant association is indicated when PRR > 0, the chi-square test value exceeds 4, and there are at least three reports of the adverse event. Higher PRR and ROR values indicate a stronger association between the drug and the AE. BCPNN and EBGM are Bayesian statistical methods that use confidence intervals to evaluate the stability and significance of estimates. BCPNN uses the confidence interval of the Information Component (IC), where an IC025 > 0 indicates a statistically significant association. EBGM05, the lower limit of the 95% confidence interval for EBGM, indicates a significant statistical association between the drug and AEs if EBGM05 > 2. The calculation methods and criteria for positive signals in disproportionality analysis are detailed in Supplementary Table S2. The overall process of this study is illustrated in Figure 1.

## 3 Results

### 3.1 Basic information on AEs of sulfasalazine

From the first quarter of 2004 to the fourth quarter of 2023, we extracted 16, 800, 135 adverse event reports from the FAERS database. Among these reports, we identified 7,156 instances where sulfasalazine was the primary drug associated with adverse reaction events. In the sulfasalazine-related adverse event reports (Supplementary Table S3), the proportion of female patients (68.06%) was significantly higher than that of male patients (26.41%). The incidence of AEs was most common in the 45–65 age group (35.03%). Analyzing the sources of reports, the majority came from physicians (38.39%). Notably, sulfasalazine was most frequently used for the treatment of rheumatoid arthritis (33.49%). Regarding severe adverse outcomes, the clinical outcomes caused by sulfasalazine mainly included other serious - Important Medical Events (51.95%), hospitalization (32.13%), life-threatening (6.06%), disability (4.55%), death (4.00%), congenital anomaly (1.00%), and required intervention to prevent permanent impairment/damage (0.31%).

### 3.2 Detection of adverse signals for sulfasalazine

Our study shows that sulfasalazine-related adverse reaction reports mainly involve 24 SOCs. The results (Supplementary Table S4) indicate that the three most frequently affected systems by sulfasalazine are general disorders and administration site conditions (n = 4,262, ROR 1.08, PRR 1.06, IC 0.09, EBGM 1.06), gastrointestinal disorders (n = 2,387, ROR 1.23, PRR 1.21, IC 0.27, EBGM 1.21), and skin and subcutaneous tissue disorders (n = 2,182, ROR 1.85, PRR 1.77, IC 0.82, EBGM 1.77). Although the EBGM values for the aforementioned three SOC categories are below 2, other signal detection algorithms, such as the ROR, PRR, and IC, offer varying degrees of risk indication. Each of these algorithms has its own strengths in signal detection, providing insights into potential risks from different perspectives. In this study, the results of some SOCs are consistent with the drug label. Notably, congenital, familial and genetic disorders, musculoskeletal and connective tissue disorders, and infections and infestations are not mentioned in the sulfasalazine drug label, indicating a need for further research and verification.

**FIGURE 2:**
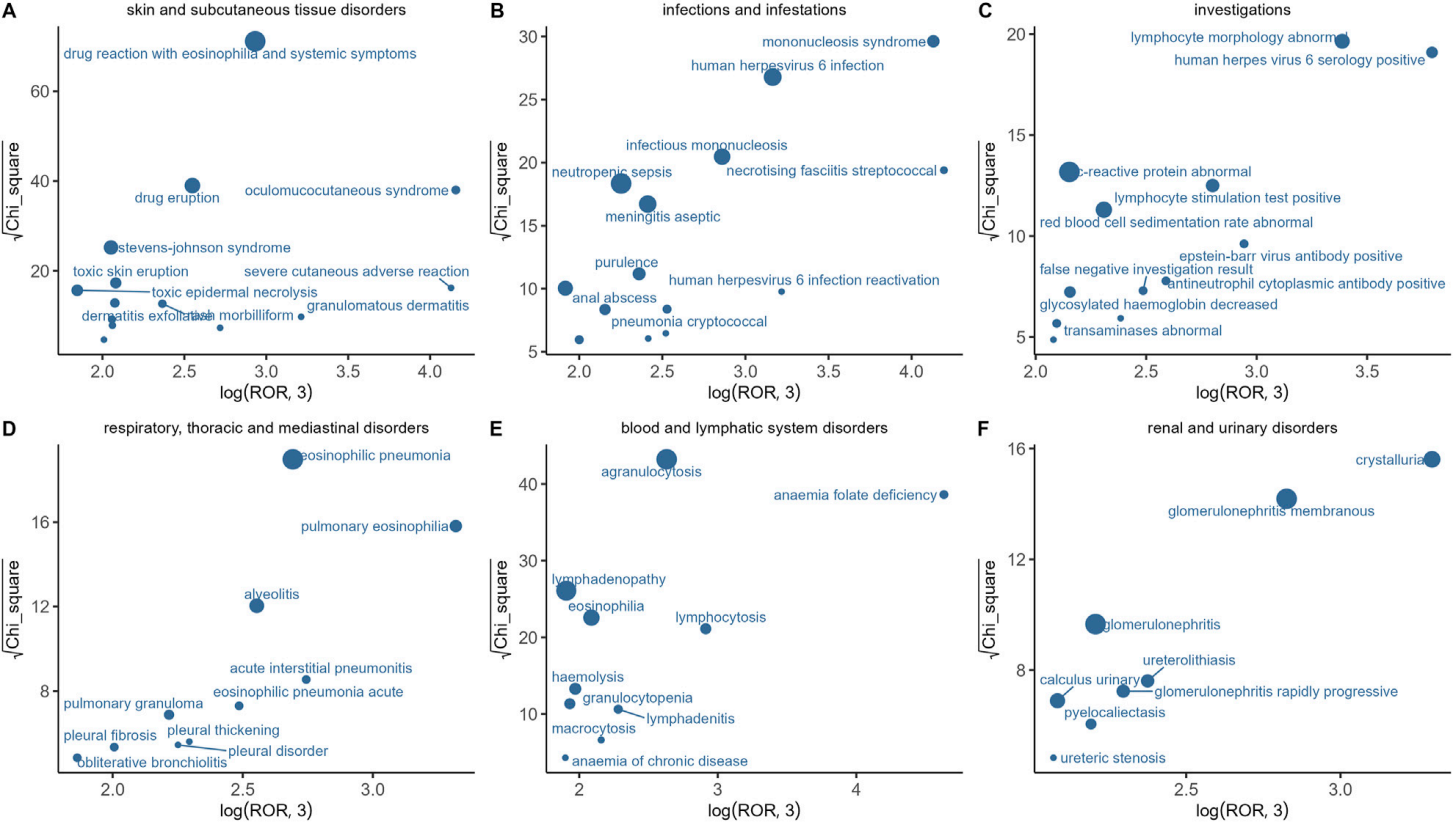
Distribution of AES among six SOCs. **(A)** Skin and subcutaneous tissue disorders; **(B)** Infections and infestations; **(C)** Investigations; **(D)** Respiratory, thoracic and mediastinal disorders; **(E)** Blood and lymphatic system disorders; **(F)** Renal and urinary system disorders.

In this study, we analyzed the AE signals of sulfasalazine across six SOCs and identified the characteristics of adverse events within each SOC (Figure 2). Using ROR and Chi-square analysis, we found that oculomucocutaneous syndrome exhibited the most significant signal among the skin and subcutaneous tissue disorders. For infectious diseases, necrotising fasciitis streptococcal and mononucleosis syndrome were identified as the primary adverse reactions. In the investigations category, human herpes virus six serology positive, abnormal lymphocyte morphology, and Epstein-Barr virus antibody positive demonstrated strong positive signal associations with sulfasalazine. Additionally, pulmonary eosinophilia and acute interstitial pneumonitis were the most prominent signal features within the respiratory system. In the blood and lymphatic system disorders, the main adverse reactions included anaemia folate deficiency, lymphocytosis, and agranulocytosis. In the renal and urinary system disorders, crystalluria and membranous glomerulonephritis were the most prominent signals.

We used four different signal detection algorithms to evaluate the strength of the association signals between the drug and adverse reaction events, ultimately identifying 101 PTs (Supplementary Table S1). Subsequently, based on the results of the ROR algorithm, we ranked these PTs and selected the top 30 PTs with the strongest signal strength (Supplementary Table S5). Among them, the top 10 PTs with the strongest signal strength are anaemia folate deficiency, necrotising fasciitis streptococcal, oculomucocutaneous syndrome, mononucleosis syndrome, teratogenicity, human herpes virus six serology positive, eosinophilic myocarditis, pulmonary eosinophilia, pleuropericarditis, and lymphocyte morphology abnormal.

## 4 Discussion

### 4.1 General analysis of ADE reports

This study analyzed post-marketing AEs associated with sulfasalazine by mining the FAERS database. As of the fourth quarter of 2023, there were 7,156 AE reports related to sulfasalazine. Clinically, sulfasalazine is primarily used to treat inflammatory diseases such as ankylosing spondylitis, Crohn’s disease, ulcerative colitis, and rheumatoid arthritis, aligning with the current indications of the drug (Plosker and Croom, 2005; Toussirot and Wendling, 2001; Zenlea and Peppercorn, 2014). The patients’ ages ranged from 18 to 65 years, with a majority being female. This higher prevalence of female patients may be attributed to the greater incidence of autoimmune diseases among women (Fish, 2008; Whitacre, 2001). Among the reporting countries, excluding those with unknown locations, the highest proportion of reports came from the United States. This was followed by Canada, Japan, the United Kingdom, and other developed countries. This distribution may be influenced by factors such as the development of the FAERS database, the level of national development, and the awareness of adverse drug reactions among the populations of these countries (Madhushika et al., 2022).

### 4.2 Skin and subcutaneous tissue disorders

Sulfasalazine is an effective anti-inflammatory drug, but it can also cause adverse reactions in the skin and subcutaneous tissue. These adverse reactions can vary widely, ranging from mild rashes and itching to severe systemic exfoliative skin reactions (Atheymen et al., 2013; Iemoli et al., 2006). The results of this study indicate that drug reaction with eosinophilia and systemic symptoms, dermatitis exfoliative, hypersensitivity vasculitis, and Stevens-Johnson syndrome are high-risk signals for sulfasalazine, consistent with common skin adverse reactions recorded in the drug label. Previous studies have reported cases of different types of severe skin reactions, including Stevens-Johnson syndrome, exfoliative dermatitis, and Drug Reaction with Eosinophilia and Systemic Symptoms (Atheymen et al., 2013; Manvi et al., 2022). The potential mechanisms of these severe skin adverse reactions mainly include metabolite-mediated cytotoxic effects and immune-mediated hypersensitivity reactions (Atheymen et al., 2013). Although the incidence of these reactions is relatively low, their serious and potentially fatal consequences require significant attention in clinical use (Tremblay et al., 2011; Teo and Tan, 2006; Girelli et al., 2013). Additionally, acute febrile neutrophilic dermatosis, oculomucocutaneous syndrome, and granulomatous dermatitis are not listed on the sulfasalazine drug label. Sweet’s syndrome is a rare inflammatory skin disease characterized by fever, elevated white blood cell count, red plaques, and neutrophilic infiltration. Clinical case reports have indicated that some patients developed Sweet’s syndrome after taking sulfasalazine, with symptoms including fever, elevated white blood cell count, and red plaques (Romdhane et al., 2016). Although the exact mechanism is not yet fully understood, it may be related to allergic reactions and neutrophil dysfunction induced by sulfasalazine and its metabolites (Romdhane et al., 2016; Yamamoto, 2014; Ayyash and Sampath, 2021).

### 4.3 Blood system disorders

Sulfasalazine can cause varying degrees of damage to the hematopoietic system, resulting in various hematologic abnormalities. The most common reported hematologic adverse reactions include granulocytopenia, folate deficiency anemia, hemolysis, and eosinophilia. Agranulocytosis is a rare but serious adverse reaction associated with sulfasalazine use, as indicated by current research (Jick et al., 1995). Sulfasalazine may inhibit granulocyte production or enhance their peripheral destruction via immune-mediated mechanisms (Dery and Schwinghammer, 1988). Furthermore, studies have identified that agranulocytosis associated with sulfasalazine is primarily linked to the MHC region on chromosome 6, which encodes HLA genes. Specifically, certain HLA alleles, such as HLA-B08:01 and HLA-A31:01, significantly increase the risk of this adverse reaction. This underscores the critical role of genetic factors in disease susceptibility and emphasizes the importance of personalized treatment strategies (Wadelius et al., 2018; Fathallah et al., 2015). Studies indicate that sulfasalazine can interfere with the absorption and metabolism of folate, and this effect is dose-dependent, with higher doses significantly increasing the risk of folate deficiency (Grindulis and McConkey, 1985). Hemolytic anemia is another potential hematologic toxicity associated with sulfasalazine use, particularly in patients with glucose-6-phosphate dehydrogenase (G6PD) deficiency. The deficiency of this enzyme makes red blood cells more susceptible to oxidative damage caused by sulfasalazine (Youngster et al., 2010; Sahm et al., 2021).

### 4.4 Infections and infestations

Sulfasalazine can impair immune system function, reducing the body’s defense against various pathogens. This increases susceptibility to bacteria, viruses, and fungi (Chiu and Chen, 2020; Andrès and Maloisel, 2008; Nasiri-Jahrodi et al., 2023). Sulfasalazine can also impair neutrophil function, leading to decreased levels of these crucial anti-infective cells in peripheral blood. This further increases the risk of infection (Andrès and Maloisel, 2008; Farr et al., 1991). In this study, positive signals were observed for infections such as streptococcal necrotizing fasciitis, human herpesvirus six infection or reactivation, pneumococcal infection, cryptococcal pneumonia, neutropenic sepsis, and latent tuberculosis. Previous studies suggest that sulfasalazine may activate the HHV-6 virus, which can trigger and exacerbate hypersensitivity syndromes in susceptible patients. This may lead to recurrent conditions and multiple drug hypersensitivity (Tan and Chan, 2016; Komura et al., 2005). This highlights the importance of closely monitoring and managing the risk of HHV-6 reactivation during sulfasalazine treatment. We also identified a potential association between sulfasalazine use and aseptic meningitis, which aligns with existing clinical case reports. Aseptic meningitis is characterized by non-infectious inflammation of the meninges. Symptoms typically include severe headache, fever, neck stiffness, nausea, and vomiting, and may also involve altered consciousness or seizures. Although relatively rare in clinical practice, aseptic meningitis warrants attention in patients undergoing sulfasalazine treatment (Yelehe-Okouma et al., 2018; Tay et al., 2012; Salouage et al., 2013).

### 4.5 Respiratory, thoracic and mediastinal disorders

Studies have demonstrated that sulfasalazine has pulmonary toxicity, directly damaging the respiratory system. Clinically, sulfasalazine-induced pulmonary toxicity manifests in various forms, commonly including interstitial lung disease, pneumonia, pulmonary fibrosis, and eosinophilic pneumonia. In this study, AEs associated with sulfasalazine included acute interstitial pneumonitis, eosinophilic pneumonia, alveolitis, pleural fibrosis, and pulmonary granuloma, consistent with previous clinical reports (Moss and Ind, 1991; Gabazza et al., 1992; Averbuch et al., 1985; Moseley et al., 1985). These conditions typically present as progressive dyspnea, cough, fever, and chest pain. In severe cases, they can lead to respiratory failure. Imaging studies, such as chest X-rays, often reveal diffuse alveolar interstitial infiltrates or patchy shadows, indicating abnormal lung patterns (Parry et al., 2002). Interstitial lung disease is a common and severe manifestation of sulfasalazine-related pulmonary toxicity. Pathological features include alveolar septal thickening, inflammatory cell infiltration, and fibrotic tissue formation. The development of interstitial lung disease may be related to the immunomodulatory effects of sulfasalazine, which can induce inflammatory cell infiltration and fibrotic reactions in the lungs, leading to tissue damage (Hamadeh et al., 1992). Sulfasalazine may also induce eosinophil accumulation in lung tissue, leading to eosinophilic pneumonia. In these cases, peripheral blood eosinophil counts are usually elevated, indicating an allergic or immune-mediated response (Nasim et al., 2021; Editorial, 1974). In clinical management, given sulfasalazine’s pulmonary toxicity, physicians should exercise heightened vigilance. If patients develop unexplained respiratory symptoms such as dyspnea, cough, and fever, the possibility of drug-induced toxicity should be considered.

### 4.6 Renal and urinary disorders

Evidence suggests that sulfasalazine may cause severe renal damage. Sulfasalazine’s nephrotoxicity is primarily mediated through oxidative stress, as evidenced by increased serum creatinine and blood urea nitrogen levels, elevated reactive oxygen species, enhanced lipid peroxidation, and glutathione depletion. Sulfasalazine is metabolized into mesalazine and sulfapyridine, both of which may contribute to renal injury. Sulfapyridine, a sulfonamide, is particularly known for its potential adverse effects on the kidneys. Renal damage caused by sulfasalazine is often irreversible, with no specific treatment currently available for sulfasalazine-induced renal injury (Linares et al., 2011; Heidari et al., 2016a). This study reports adverse effects on the renal and urinary systems, primarily including crystalluria, membranous glomerulonephritis, ureteral stones, and glomerulonephritis. Current research primarily focuses on sulfasalazine’s impact on renal function and interstitial damage, with little direct evidence of glomerular injury, indicating a need for further verification. Additionally, studies have shown that sulfasalazine’s metabolite sulfapyridine is excreted in the urine. When the drug highly concentrates in the renal tubules and collecting ducts, it may form crystals. These crystals are more likely to form under urine supersaturation, particularly in conditions of reduced body fluids or low urine pH, leading to tubular obstruction and crystalluria, which may aggravate renal damage and potentially cause anuric renal failure (Perazella and Rosner, 2022; Durando et al., 2017). Therefore, for patients receiving sulfasalazine treatment, particularly those with a history of kidney stones, renal insufficiency, or dehydration, timely renal function monitoring and preventive measures are crucial (Durando et al., 2017).

### 4.7 Other controversial and new adverse reactions

Assessing the teratogenic risks of sulfasalazine during pregnancy is complex and highly debated. Nørgård et al. conducted a study to evaluate the teratogenic risks of sulfasalazine during pregnancy. They used a case-control study design, analyzing data from the Hungarian Congenital Abnormality Registry, which included 22,865 newborns or fetuses with congenital anomalies (case group) and 38,151 without congenital anomalies (control group). The results showed that the incidence of congenital anomalies in pregnant women treated with sulfasalazine was not significantly higher than in those who were not treated. However, the limited data necessitates further studies to completely rule out its teratogenic effects (Nørgård et al., 2001). Notably, our study indicates a strong positive signal between sulfasalazine and teratogenicity. Experimental studies have shown that sulfasalazine may cause skeletal abnormalities and cleft palate in the fetuses of pregnant mice and rats (Kato, 1973). Previous clinical case reports documented two pregnant women with inflammatory bowel disease who were treated with sulfasalazine during pregnancy, and three newborns were observed with major congenital anomalies (Newman and Correy, 1983). A study aimed to analyze the medication patterns of women and men using antirheumatic drugs before and during pregnancy in Norway and investigate the association between these medications and the risk of congenital anomalies in infants. The study found that five children whose mothers used sulfasalazine within 3 months before or during pregnancy were born with congenital anomalies (Viktil et al., 2012). A meta-analysis on the use of 5-ASA drugs in pregnant women with IBD found an association with an increased risk of congenital anomalies, although this risk increase does not exceed 1.16 times. However, this risk increase is not statistically significant (Rahimi et al., 2008). Therefore, the potential teratogenic risks of sulfasalazine to the fetus require further investigation.

It is well known that sulfonamides have hepatotoxic effects, and liver injury induced by sulfasalazine is a serious AE associated with its clinical use. Although the exact mechanism is not fully understood, defects in cellular defense functions and oxidative stress likely play significant roles. Excessive accumulation of reactive oxygen species can lead to organ dysfunction and parenchymal damage (Linares et al., 2011; Heidari et al., 2016b). Sulfasalazine-induced liver injury is mainly characterized by elevated transaminases, increased bilirubin levels, and hepatocellular necrosis. In severe cases, it may progress to granulomatous hepatitis or cholestatic liver cirrhosis (Heidari et al., 2016b; Linares et al., 2009). Granulomatous hepatitis is a specific type of liver damage typically associated with immune reactions. Sulfasalazine may induce the aggregation of inflammatory cells in the liver, leading to granuloma formation by affecting the immune system (Namias et al., 1981; Fich et al., 1984). Notably, in this study’s adverse event reports, hepatosplenic T-cell lymphoma showed a strong positive signal. Although IBD itself is not considered a risk factor for gastrointestinal lymphoma, long-term use of immunosuppressants and TNF inhibitors may increase the risk of lymphoma (Thai and Prindiville, 2010; Kotlyar et al., 2011). Suzuki et al. reported a case of a patient with UC who had been treated with 5-ASA for an extended period and eventually developed diffuse large B-cell lymphoma (DLBCL), leading to intestinal perforation. This case emphasizes that lymphoma can occur in IBD patients even without the use of traditional immunosuppressants. Although the case did not specifically mention HSTCL, we know that HSTCL is indeed associated with IBD, particularly in patients using certain treatments, suggesting that the underlying disease itself may to some extent increase the risk of lymphoma (Suzuki et al., 2021; Ashrafi et al., 2014). The results of this study indicate a positive signal association between sulfasalazine and HSTCL, challenging the previous view that HSTCL is primarily associated with the use of thiopurines and TNF inhibitors. This suggests that sulfasalazine, a common drug in the treatment of IBD, may have a potential link with the occurrence of HSTCL. However, there is currently no clear evidence or reports directly linking it to the occurrence of HSTCL, so more research is needed to establish a causal relationship.

## 5 Limitations

FAERS is a pharmacovigilance system that relies primarily on voluntary reports, which presents inherent limitations in extracting accurate adverse drug reaction information. Firstly, FAERS data predominantly originate from voluntary reports, which may introduce biases in both the quantity and quality of the reports. This bias can result in an incomplete representation of the adverse reactions experienced by all patients. Secondly, voluntary reports often lack standardized criteria, leading to incomplete or inaccurate information. This lack of standardization affects the overall reliability of the data. Moreover, subjective judgment by reporters can lead to misreporting or underreporting, further affecting the outcomes of data analysis. Therefore, cross-validating adverse drug reactions with other pharmacovigilance databases, such as VigiBase and Canada Vigilance, is crucial. These databases offer alternative reporting sources and standards that can complement and validate findings from FAERS data, thereby enhancing the accuracy and reliability of drug safety assessments. Furthermore, conducting additional prospective studies and randomized controlled trials is essential for validating potential associations identified in FAERS and clarifying causal relationships. This approach ensures a comprehensive and scientific evaluation of drug safety.

## 6 Conclusion

This study conducted a systematic analysis of AEs related to sulfasalazine using the FAERS database. The analysis revealed potential serious risks associated with the drug across multiple organ systems. Despite being a widely used anti-inflammatory medication, sulfasalazine is associated with a range of complex adverse reactions. Significant risk signals were identified particularly in the skin, hematologic, infectious, respiratory, and urinary systems. Sulfasalazine has been linked to severe adverse reactions in the skin and subcutaneous tissues, such as Stevens-Johnson syndrome and exfoliative dermatitis. Hematologic adverse reactions associated with sulfasalazine include agranulocytosis and folate deficiency anemia. Moreover, sulfasalazine may increase the risk of infections, as indicated by significant signals for severe conditions such as neutropenic sepsis and cryptococcal pneumonia. In the respiratory system, sulfasalazine may lead to pulmonary toxicities, such as interstitial lung disease and eosinophilic pneumonia. Similarly, sulfasalazine’s nephrotoxic effects, including crystalluria and glomerulonephritis, require close attention. The study also suggests a potential teratogenic risk associated with sulfasalazine use during pregnancy; however, this finding requires further validation. Furthermore, the potential association between sulfasalazine and hepatosplenic T-cell lymphoma requires further investigation. In conclusion, this study highlights the serious risks of adverse reactions associated with sulfasalazine across multiple systems and emphasizes the need for vigilant monitoring in clinical practice. Future research should focus on elucidating the mechanisms underlying these adverse reactions, validating the identified risks through large-scale prospective studies, and developing personalized treatment strategies for high-risk populations to improve drug safety management.

## Data Availability

The original contributions presented in the study are included in the article/Supplementary Material, further inquiries can be directed to the corresponding authors.
